# First Complete Genome Sequence of a Feline Morbillivirus Isolate from Germany

**DOI:** 10.1128/genomeA.00244-18

**Published:** 2018-04-19

**Authors:** Michael Sieg, Annett Vahlenkamp, Christoph Georg Baums, Thomas Wilhelm Vahlenkamp

**Affiliations:** a Institute of Virology, Faculty of Veterinary Medicine, University of Leipzig, Leipzig, Germany; b Veterinary Practice Dr. Gorzny & Partner Tierärzte, Markranstädt, Germany; c Institute for Bacteriology and Mycology, Faculty of Veterinary Medicine, University of Leipzig, Leipzig, Germany

## Abstract

The first cell culture isolation and whole-genome sequence of a feline morbillivirus from Germany are described here. Phylogenetic analysis revealed highest similarity to isolates from Japan and a more distant relationship to strains from Italy, Hong Kong, and the United States. Therefore, feline morbilliviruses should be divided into two different genotypes.

## GENOME ANNOUNCEMENT

Feline morbilliviruses (FmoPVs) are enveloped, single-stranded RNA viruses belonging to the family Paramyxoviridae and were first discovered in stray cats from Hong Kong in 2012 (1). Since then, FmoPVs have been shown to be prevalent in other countries, including Japan (2), the United States (3), Turkey (4), Brazil (5), Thailand (6), Italy (7), and Germany (8). Only a limited number of viral isolates and whole-genome sequences are available. Here, were report the first cell culture isolation and complete genome sequence of an FmoPV isolate from Germany.

The sample was taken from a 15-year-old male cat suffering from polyuria-polydipsia syndrome. The presence of FmoPV RNA within the urine was detected as described previously (8). Further urine analysis showed proteinuria and bacteriuria caused by a mixed infection with Escherichia coli and Klebsiella pneumoniae subsp. pneumoniae. Whereas the presence of FmoPV RNA persisted for several months, bacteriuria was cleared shortly after the onset of antimicrobial therapy. The urine sample was inoculated onto CrFK cells as described previously (1) and incubated for 7 days until further passaging. Supernatant from the fourth passage was FmoPV-positive in PCR analysis, and the infected cell monolayer stained positive in an immunofluorescence assay using an FmoPV nucleoprotein-specific antibody. Viral RNA was extracted from the cell culture supernatant using the QIAamp viral RNA minikit (Qiagen), and the whole genome of the isolated strain was amplified by using the SuperScript III One-Step Real-Time PCR System with Platinum *Taq* High Fidelity (Life Technologies). For this purpose, degenerate primers were designed based on available whole-genome sequences from feline morbilliviruses in GenBank. Sequencing was done by using Sanger’s dideoxy termination method in duplicates. The resulting nucleotide sequences were assembled by homology screening with the FmoPV strain M252A (GenBank accession no. JQ411016). For phylogenetic analysis, we calculated genetic distances employing the Tamura-Nei model at the nucleotide level.

The whole-genome sequence of the FmoPV isolate designated TV17 was 16,050 nucleotides in length and complied with the “rule of six” characteristic for paramyxoviruses. Six open reading frames (3′-NP-P/V/C-M-F-H-L-5′) were annotated, as shown for previously described feline morbilliviruses (1). Phylogenetic analysis of the whole-genome sequence showed highest nucleotide similarity (93.8%) to Japanese strains SS1 (GenBank accession no. AB910309) and OtJP001 (GenBank accession no. AB924120). In contrast, homologies to the Hong Kong strain M252A (GenBank accession no. JQ411016), the Italian strain Piuma/2015 (GenBank accession no. KT825132), and the U.S. strain US1 (GenBank accession no. KR014147) were only 88.2%, 88.0% and 87.8%, respectively. Comparison of the amino acid sequences between TV17 and SS1 revealed 98%, 92%, 98%, 97%, 96%, and 98% similarity of the nucleoprotein, the phosphoprotein, the matrix protein, the fusion protein, the hemagglutinin protein, and the RNA polymerase, respectively.

Together with the findings from another workgroup in Japan (9), our results suggest that feline morbilliviruses should be divided into two different genotypes.

### Accession number(s).

The complete sequence of the feline morbillivirus isolate TV17 is available in GenBank under the accession no. MG563820.
